# Integrating Quality Management and Male Reproductive Health in Assisted Reproduction

**DOI:** 10.1590/S1677-5538.IBJU.2025.0180

**Published:** 2025-04-10

**Authors:** Fabiola C. Bento, Rita C. S. Figueira, Sandro C. Esteves

**Affiliations:** 1 Clínica de Andrologia e Reprodução Humana ANDROFERT Campinas SP Brasil ANDROFERT, Clínica de Andrologia e Reprodução Humana, Campinas, SP, Brasil; 2 Universidade Estadual de Campinas - UNICAMP Disciplina de Urologia Departamento de Cirurgia Campinas SP Brasil Departamento de Cirurgia, Disciplina de Urologia, Universidade Estadual de Campinas - UNICAMP, Campinas, SP, Brasil

**Keywords:** Infertility, Male, Reproductive Techniques, Assisted, Sperm Injections, Intracytoplasmic

## Abstract

Quality management is essential to ensure consistent, safe, and effective outcomes in assisted reproductive technology (ART) centers. However, traditional quality assessments often overlook male infertility, which contributes to nearly half of all infertility cases. This article explores the implementation of a quality management system (QMS), specifically ISO 9001, tailored to ART centers that prioritize male reproductive health. Drawing from our experience at ANDROFERT, a male-focused fertility clinic, we demonstrate how process standardization, structured workflows, and continuous improvement strategies can optimize diagnostics, microsurgical procedures, and laboratory practices. Integrating male-specific procedures—such as varicocele repair, microdissection testicular sperm extraction (micro-TESE), and vasovasostomy—into the QMS is discussed, along with performance monitoring tools, including key performance indicators and patient satisfaction metrics. Collaboration with an academic institution is essential to support education and ensure training is aligned with quality and safety protocols. Our center's dual IVF laboratories and andrology services exemplify how advanced testing, including sperm DNA fragmentation analysis and handling of surgically retrieved sperm, are seamlessly integrated into quality pathways. By embedding male reproductive health into QMS frameworks, ART centers can improve clinical outcomes, foster interdisciplinary collaboration, and enhance patient engagement. We advocate for a multidimensional approach to quality—beyond pregnancy rates—encompassing safety, effectiveness, patient-centeredness, timeliness, efficiency, and equity. This model strengthens clinical performance and ensures sustainable, evidence-based fertility care for male patients.

## INTRODUCTION

In healthcare, "quality" is often defined as the degree to which services meet established standards and fulfill patient expectations. While this concept is inherently subjective, effective quality management requires objective, measurable indicators. In the context of assisted reproductive technology (ART), pregnancy rates are frequently used as a proxy for success. However, such a narrow focus can be misleading. A high pregnancy rate does not necessarily reflect superior care, particularly when essential factors such as patient selection, case complexity, and individualized treatment options are not accounted for.

For instance, an ART program reporting a 50% pregnancy rate may not be inherently superior to one reporting 30%. The difference may stem from varied patient demographics, the use of gamete donation, number of embryos transferred, or exclusion of poor-prognosis cases. Moreover, aggressive clinical strategies can inflate outcome metrics while compromising patient-centered care.

In this context, quality management in ART centers should emphasize process-based approaches, where outcomes are evaluated not solely by clinical success, but by how well systems and workflows support safe, equitable, and patient-focused reproductive care. This is especially crucial in the treatment of male infertility, a field that requires integration of urological expertise with embryology, andrology, and laboratory standards (1-4).

## ASSESSING QUALITY IN ART CENTERS

Quality in ART centers must be evaluated through the lens of structured process management. A Quality Management System (QMS), such as ISO 9001, provides a formal framework to ensure that service delivery is systematic, measurable, and continuously improving.

**Figure 1:**
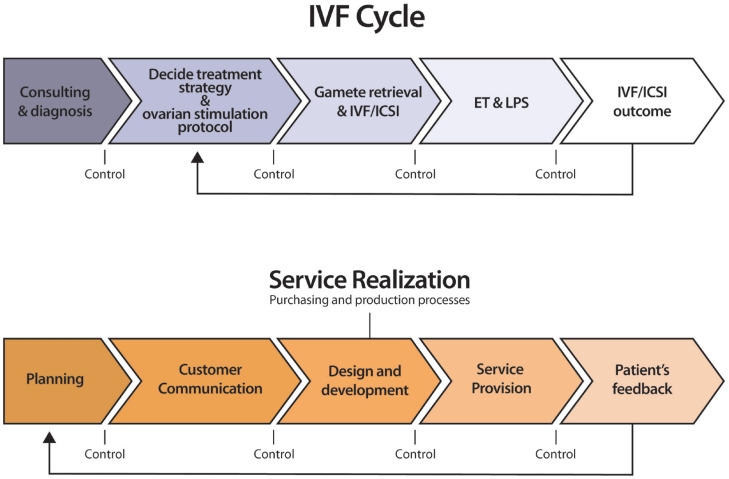
Flowchart of mapping procedure applied to the in vitro fertilization (IVF) treatment cycle.

At its core, quality is about how well the process transforms inputs—staff expertise, equipment, laboratory protocols—into safe, effective, and timely care. ART centers are particularly well-suited for QMS implementation, as nearly all clinical and laboratory procedures are repeatable and protocol-driven, making them amenable to standardization and performance evaluation. Figure-1 illustrates how ART activities can be mapped to a process-based quality system.

IVF = In vitro fertilization; ICSI = intracytoplasmic sperm injection; ET = embryo transfer; LPS = luteal phase support

## QUALITY INDICATORS IN INFERTILITY CARE

Quality indicators are measurable elements used to assess performance and are essential in managing ART programs (5). While success rates remain important, a more holistic approach is needed—particularly for male infertility care, where outcomes depend not only on fertilization or embryo transfer but also on appropriate diagnosis, sperm retrieval techniques, DNA integrity assessment, and individualized management. Most IVF quality indicators emphasize effectiveness and safety (6-14).

The Institute of Medicine (IOM) outlines six dimensions of healthcare quality that should guide ART practice (15): i. Effectiveness, ii. Safety, iii. Efficiency; iv. Timeliness, v. Equity, and vi. Patient-centeredness. Most current ART quality indicators emphasize safety and effectiveness. Yet, evidence from patient-centered research shows that attributes like clear communication, emotional support, and individualized care are equally important.

In a Delphi study across two European countries, Dancet et al. identified 24 quality indicators in fertility care, highlighting safety, effectiveness, and patient-centeredness as top priorities (16). Interestingly, while clinicians focused on measurable outcomes, patients consistently emphasized interpersonal aspects of care—such as emotional support and personalized communication. Cai et al. later confirmed these findings in a large Chinese multicenter survey, showing a consistent divergence between patients’ and providers’ definitions of "quality" (17). This underscores the need for ART centers—particularly those focused on male reproductive health—to align their services not just with technical standards, but with patient expectations (Table-1).

**Table 1:** Quality dimensions and indicators in infertility care per consensus between healthcare professionals and patients. Safety, effectiveness, and patient-centeredness were considered the most critical dimensions in an iterative Delphi survey.

Quality Dimension	Quality Indicator	Type of Indicator
Safety	Hospitalization due to severe complications (e.g., OHSS, infection, bleeding, pain) per total ART cycles.	Outcome
Safety	Complications per total fresh ART cycles.	Outcome
Safety	Gamete/embryo loss due to error per total MAR cycles.	Process
Safety	Reported incidents by care providers per treatment cycles.	Process
Effectiveness	Live birth per total treated patients.	Outcome
Effectiveness	Live birth after ≤3 fresh ART cycles per patients starting ART.	Outcome
Effectiveness	Live birth per fresh ART cycle with embryo transfer.	Outcome
Effectiveness	Pregnancy rate in women <36 years per fresh ART cycle.	Outcome
Patient-centeredness	Offer of psychosocial counseling per total patients.	Process
Patient-centeredness	Multidisciplinary meetings to discuss psychosocial context.	Structural
Patient-centeredness	Availability of counseling at critical points.	Structural
Patient-centeredness	Patients felt heard per total surveyed.	Process
Efficiency	Diagnosis completion before MAR per patients starting MAR.	Process
Efficiency	Clinic website with basic info and FAQs available.	Structural
Efficiency	Electronic records with clinical data and reporting tools.	Structural
Efficiency	FTE care providers per treated patient per provider type.	Structural
Timeliness	Waiting time from request to first appointment.	Process
Timeliness	Waiting time for urgent consults during MAR.	Process
Timeliness	Time from first appointment to treatment start.	Process
Timeliness	Waiting room time before consultation begins.	Process
Equity	Patients felt respected by physician per surveyed.	Process
Equity	Clinic criteria for MAR access clearly described.	Structural
Equity	Clear ethical limitations policy at all times.	Structural
Equity	Protocols follow international equity guidelines.	Structural

ART – Assisted Reproductive Technology; OHSS – Ovarian Hyperstimulation Syndrome; MAR – Medically Assisted Reproduction; FTE – Full-Time Equivalent; FAQ – Frequently Asked Questions

Outcome indicators reflect the results of care delivered to patients. Process indicators assess actions taken during care delivery, reflecting what healthcare providers do for patients. Structural indicators describe the environment in which care is provided, including infrastructure, staffing, equipment, and organizational frameworks. Adapted from Dancet et al., Human Reproduction 2013;28(6):1584–97, with permission from Oxford University Press

## INTEGRATING MALE REPRODUCTIVE HEALTH INTO QUALITY MANAGEMENT

Although the burden of infertility is equally shared by male and female partners, male factor infertility has historically received less attention in ART programs (18). Yet, it contributes to nearly half of all infertility cases, either as a primary or coexisting diagnosis (1-4). A high-performing ART center must therefore have structured processes to diagnose, evaluate, and manage male infertility within its quality framework.

Quality management in this context requires four essential commitments: i. Comprehensive and timely male evaluation, not limited to basic semen analysis but incorporating advanced semen analysis, hormonal profiling, genetic testing, imaging, and testicular biopsy, ii. Surgical treatment, iv. Pharmacological treatment, and v. Sperm retrieval techniques (6-14, 19-29). Close integration of andrology with embryology and clinical care, enabling individualized sperm selection methods, DNA fragmentation testing, and advanced laboratory when appropriate (6-14, 30).

Collaboration with urologists specializing in male reproduction, ensuring that patients receive surgical or medical interventions that can improve outcomes or avoid unnecessary ART cycles. By embedding these elements into the QMS, ART centers can avoid a common pitfall: overlooking the male partner or treating him as merely a sperm provider, rather than an integral part of the reproductive process. This is especially important given the rising awareness of how paternal factors—including age, comorbidities, and sperm DNA quality—affect embryo development, pregnancy rates, and long-term offspring health (12, 13, 20, 23, 31).

## ADDRESSING PATIENT RETENTION AND MALE ENGAGEMENT IN ART

Another key aspect of quality care is patient retention, which is often undermined by emotional distress, misinformation, or a sense of exclusion—particularly among male partners. Men frequently report feeling sidelined during fertility treatment, with little direct communication from clinical staff and minimal psychosocial support (1, 2).

A systematic review by Gameiro et al. identified the major drivers of ART discontinuation as treatment burden, emotional strain, and perceived lack of support. For male patients, the stigma surrounding infertility and reluctance to seek urological care further complicate engagement (32).

A robust QMS can mitigate this by i. Implementing patient-centered protocols that include the male partner from the initial consultation onward, ii. Monitoring dropout rates by gender and tracking underlying reasons to inform targeted interventions, and iii. Integrating psychological support services tailored to male patients, such as counseling for azoospermia or erectile dysfunction in the context of ART. These strategies not only improve the quality of care but also optimize outcomes by maintaining continuity and compliance throughout treatment.

## ESTABLISHING A QMS WITH A MALE REPRODUCTIVE HEALTH FOCUS

Implementing a Quality Management System like ISO 9001 provides an excellent framework for integrating male reproductive health into the fabric of an ART center. At our institution, the QMS was adapted to address specific male-focused priorities, including (33):

1. Development of clinical protocols for the evaluation and treatment of non-obstructive azoospermia, including indications for hormonal therapy, varicocele repair, and sperm retrieval procedures.
2. Standardized sperm handling and cryopreservation standard operating procedures (SOPs), accounting for testicular versus ejaculated samples and sperm DNA integrity testing.
3. Metrics for embryologist performance that differentiate outcomes by sperm origin (e.g., testicular vs. epididymal vs. ejaculate) and morphology.
4. Physician-specific KPIs that reflect proficiency in counseling and managing male infertility cases, especially complex or surgical cases.

## EMBEDDING MALE INFERTILITY PROCEDURES INTO QMS

A comprehensive ART program must not only evaluate male infertility but also be equipped to treat it. Male reproductive surgeries, both for fertility restoration and sperm retrieval, are integral to this care pathway. These procedures demand surgical precision, coordinated lab-clinic workflows, and postoperative outcome tracking—making them ideal candidates for quality standardization under a QMS.

At our center, a full spectrum of male infertility procedures is offered (9, 10-12, 14,19-22, 26-29, 34), including microsurgical varicocele repair, aimed at improving semen quality or enabling sperm retrieval in men with non-obstructive azoospermia (NOA), microsurgical sperm retrieval techniques such as MESA (Microsurgical Epididymal Sperm Aspiration) and micro-TESE (Microsurgical Testicular Sperm Extraction), particularly critical in cases of NOA or obstructive azoospermia where ejaculation is absent or unproductive. Reconstructive surgeries, such as vasovasostomy and vasoepididymostomy, for men with prior vasectomy or post-infectious obstructions, restoring natural fertility without resorting to IVF. Office-based or minimally invasive procedures, including TESA (Testicular Sperm Aspiration) and PESA (Percutaneous Epididymal Sperm Aspiration), used strategically in obstructive cases or as salvage techniques.

These interventions are fully integrated into our QMS protocols, from patient selection to surgical planning, lab coordination, and postoperative follow-up. Specific SOPs ensure standardization across i. Patient counseling and informed consent, especially in complex surgical cases or when fertility preservation is needed; ii. Scheduling and communication between surgical teams and embryologists, particularly when sperm retrieval must be synchronized with oocyte collection; iii. Cryopreservation protocols for surgically retrieved sperm, often limited in number and quality, requiring strict handling and storage parameters.

## TRAINING THE NEXT GENERATION: COLLABORATION WITH UNICAMP

As part of our institutional mission, our center maintains a long-standing collaboration with the Division of Urology at the University of Campinas (UNICAMP), a recognized center of excellence in academic urology. Senior-year urology residents rotate through our clinic, gaining hands-on exposure to male reproductive surgery, andrology diagnostics, and ART-based fertility preservation.

Incorporating trainees into a highly regulated environment presents unique challenges, particularly in a setting governed by a QMS. To accommodate this, we have developed dedicated protocols and supervision pathways to ensure that (i) Clinical activities involving residents are aligned with safety, efficiency, and consistency standards, (ii) Training milestones are tracked and assessed within the QMS framework, using checklists and feedback mechanisms, (iii) Access to procedures and labs is regulated, with tiered responsibilities based on training level and direct mentorship by senior faculty.

This dual commitment to quality care and academic development reinforces our institutional culture, ensuring that teaching does not compromise patient outcomes but instead enhances team performance and system robustness.

## ANDROLOGY AND IVF LAB CAPABILITIES WITHIN QMS

High-quality management of male infertility extends beyond the surgical suite. Our andrology laboratory offers a full suite of diagnostic services, including (i) Standardized semen analysis following WHO criteria, (ii) Advanced sperm function testing, such as sperm DNA fragmentation assays, essential in evaluating unexplained infertility, recurrent IVF failure, and guiding male infertility treatment, and (iii) Specialized preparation techniques for surgically retrieved or severely impaired sperm samples.

Our two IVF laboratories are fully equipped and experienced in handling sperm samples from male infertility patients. This includes (i) Processing micro-TESE specimens, which often involve sparse, immotile sperm requiring careful identification and isolation, (ii) Applying advanced techniques like ICSI, oocyte and sperm activation protocols, sperm vitrification, and preimplantation genetic testing. Using strict chain-of-custody and documentation protocols for sample identification, tracking, and performance benchmarking.

Both labs operate under SOPs aligned with ISO 9001 and national regulations, ensuring procedural consistency and minimizing inter-laboratory variation. Lab staff receive continuous training, and each embryologist's performance is monitored via key performance indicators (KPIs) tailored to complex male infertility cases.

## ADDRESSING QUALITY DIMENSIONS IN INFERTILITY CARE

ART centers must rethink how they deliver services to address all quality dimensions and objectively assess quality with evidence-based indicators. This is where a QMS becomes essential (33).

A QMS, such as ISO 9001, is highly adaptable to ART centers and offers numerous benefits, including:

1. Establishing quality objectives.
2. Organizing workflows.
3. Defining processes, procedures, and responsibilities.
4. Reducing errors and ensuring safety.
5. Monitoring performance and fostering continuous improvement.

While various QMS models are available in healthcare, ISO 9001 is the most recognized international quality management standard. Numerous ART centers globally have adopted ISO 9001 specifications. Although formal certification is not mandatory, some centers including ours, have obtained accreditation to meet regulatory demands (33, 35-39).

## REGULATORY REQUIREMENTS

In addition to meeting all technical requirements for operation, regulatory agencies in many countries mandate the implementation of a documented QMS. In Brazil, the current Collegiate Board Resolution (RDC) No. 771, issued by ANVISA (the National Agency for Sanitary Surveillance) in December 2022, outlines essential components for a comprehensive QMS. These include (39):

1. Development and Continuous Revision of SOPs: Standard Operating Procedures must be regularly reviewed and updated to reflect current practices and regulations.
2. Ongoing Personnel Training: Structured, periodic training programs are required to ensure that staff remain current with procedural updates and technological advancements.
3. Internal Audits: Regular audits must be conducted to verify compliance with technical standards and to identify areas for improvement.
4. Error and Nonconformity Management: A formal system must be in place to detect, document, address, and prevent errors and nonconformities, thereby improving process reliability and overall quality.
5. Biosafety Compliance: Strict adherence to biosafety regulations is essential to safeguard both public health and the environment.
6. Evaluation and Maintenance of Equipment and Materials: A systematic approach must be adopted to monitor the performance and upkeep of equipment and materials, ensuring operational efficiency and consistency.

Regulatory agencies also require additional components as part of a comprehensive QMS, including the development of a Quality Manual. This document must outline the following elements:

1. Quality Assurance (QA) Registry: A centralized system for documenting and managing all quality assurance activities.
2. Identification of Quality Processes: A detailed mapping of key processes, including the definition of performance metrics, establishment of benchmarks, and identification of critical control points.
3. Provision of Infrastructure and Resources: Assurance that the necessary infrastructure, equipment, and qualified personnel are available to support the implementation and maintenance of quality standards.
4. Code of Ethics and Professional Conduct: Clearly defined ethical principles and behavioral standards to be followed by all individuals involved in quality-related activities.

## IMPLEMENTING A QMS IN ART CENTERS: A PROCESS-DRIVEN APPROACH

**Figure 2:**
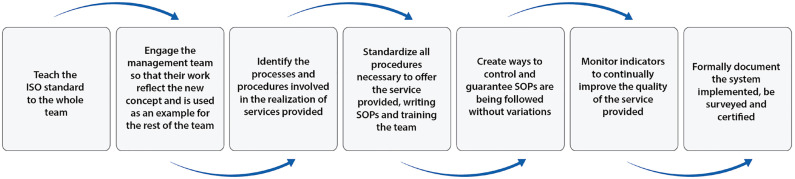
Flowchart depicting the steps taken for QMS implementation at ANDROFERT.

While various QMS models exist in healthcare, ISO 9001 remains the most widely recognized international standard (19-24). Its flexibility allows ART centers to define and control complex clinical, surgical, and laboratory workflows while ensuring compliance, traceability, and continuous improvement. At our center, implementation followed a phased, in-house strategy that allowed for deep internalization of principles and staff ownership of processes (33, 39) (Figure-2).

The key Steps in QMS implementation are detailed below.

### Step 1: Defining the Center's Mission Statement

A mission statement articulates the center's core purpose, detailing its patient population, services, and strategy for attaining excellence. It must also comply with external requirements, such as regulatory frameworks and professional guidelines.

For example, our ART center's mission is:

"To assist couples facing fertility challenges, especially those dealing with male factor infertility, by providing expert counseling, diagnosis, and treatment. We are dedicated to offering state-of-the-art facilities and technological resources to support assisted reproductive techniques while ensuring the highest standards of care."

### Step 2: Establishing a Quality Management Focus

A strong QMS requires clear quality policies, objectives, and indicators to measure performance.

- Quality Policy: A concise statement outlining management's commitment to quality. At ANDROFERT, our quality policy consists of three pillars:
  1. Ensuring patient satisfaction.
  2. Promoting professional development for staff.
  3. Continuously improving service quality.
- Quality Objectives and Indicators: These provide measurable benchmarks for assessing performance. For example, our IVF laboratory evaluates each embryologist based on fertilization rates, embryo development success, and pregnancy outcomes following embryo transfer. Additionally, we track pregnancy rates for each physician, with ongoing evaluations to maintain high clinical standards.

### Step 3: Standardizing Processes and Procedures

A fundamental aspect of QMS is identifying, documenting, and standardizing processes and procedures.

- Standard Operating Procedures (SOPs): These documents ensure that all staff members perform tasks consistently, reducing variability and improving outcomes. SOPs are crucial in ART, where complex procedures require precision and reproducibility.
- SOPs should be periodically reviewed and updated to incorporate new knowledge, identify potential errors, and enhance efficiency. A well-structured SOP ought to address three key clinical questions:
  1. Does it address a specific clinical issue?
  2. Does it guide decision-making?
  3. Does it provide clear steps for handling critical scenarios?
- Document Control: A structured document management system is essential for updating protocols and ensuring compliance. For instance, providing all staff access to updated digital SOPs reduces dependence on obsolete printed versions. At ANDROFERT, all documents are digitally stored on a secure server, with access restricted to the most recent approved versions to prevent errors from using outdated protocols.

### Step 4: Resource and Staff Management

Effective resource management is essential for sustaining a high-performing QMS.

- Human Resources: Employees should have a clearly defined job description and competency framework. Training must be offered during onboarding and at regular intervals to reinforce best practices.
- Infrastructure and Equipment: Employees must have the essential tools, training, and technology to effectively fulfill their roles. Ongoing Staff Development: Our ART Center prioritizes continuous education through structured training programs that cover technical competencies and ethical considerations in patient care.

### Step 5: Managing Errors and Non-Conformities

**Figure 3:**
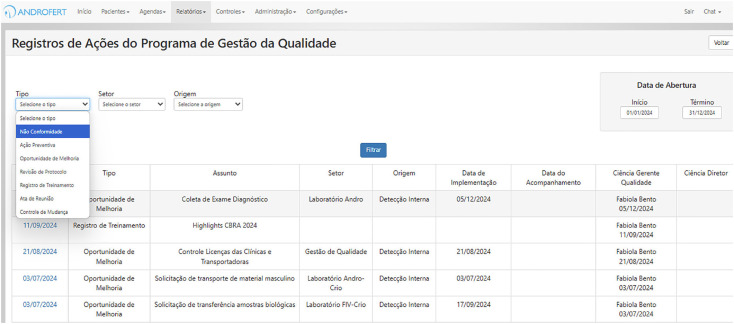
Customized Clinisys Interface for Quality Management Actions at ANDROFERT.

Error tracking and corrective action plans are integral to maintaining quality (Figure-3).

A customized version of the ClinisysIVF® software supports our comprehensive Quality Management program. The platform includes multiple tools that allow staff to log in and initiate quality-related actions across various categories, such as non-conformities, opportunities for improvement, preventive actions, training reports, and change control. When a user selects a category, a tailored form opens to begin the process. The staff can also designate relevant team members to be notified or to contribute additional information, ensuring efficient communication and coordinated follow-up across the organization.

- Non-Conformity Reporting: Any deviation from established protocols must be documented, analyzed, and addressed. Staff members are encouraged to report errors without fear of repercussions, fostering a culture of transparency and continuous learning.
- Root Cause Analysis: Corrective actions are implemented for every reported issue to prevent recurrence. For instance, additional training or workflow adjustments may be necessary if an embryologist repeatedly fails to meet fertilization rate targets.
- Preventive Actions and Continuous Improvement: Employees are encouraged to recommend preventive measures to mitigate potential risks before they affect clinical outcomes. At ANDROFERT, we maintain a database where staff can suggest workflow improvements, equipment usage, or patient care protocols.

### Step 6: Monitoring and Auditing

**Figure 4:**
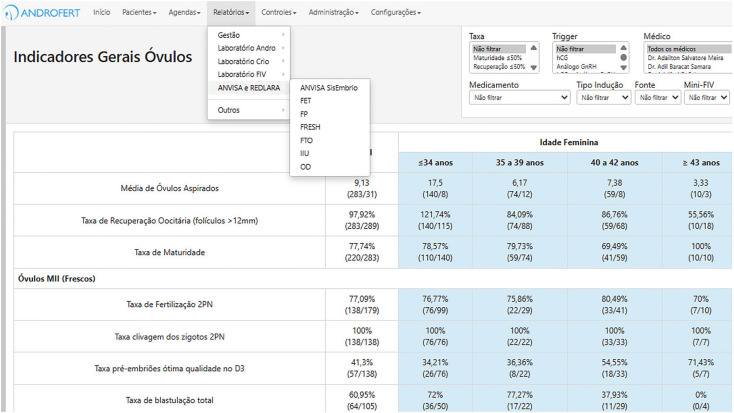
Real-Time KPI Monitoring Through the Customized ClinisysIVF® Dashboard at ANDROFERT.

Continuous monitoring is essential to verify that the QMS functions as intended. Key actions include (Figure-2):

- External Audits: Annual third-party audits ensure compliance with ISO standards.
- Internal Audits: Trained internal auditors carry out routine evaluations to confirm protocol compliance. Employees receive prior notification, and the main goal is to ensure adherence rather than penalize non-conformities.
- Key Performance Indicators (KPIs): Ongoing laboratory and clinical performance evaluations inform strategic decision-making (Figure-4).

The Clinisys IVF software at ANDROFERT includes a robust module for tracking Key Performance Indicators (KPIs), forming an essential component of our Quality Management strategy. The interface allows managers to generate customized reports in real-time, selecting specific time intervals and applying filters such as female age, treatment type, ovarian stimulation protocol, trigger method, sperm source for IVF/ICSI, responsible doctor, or the incubator used for embryo development. This functionality enables continuous monitoring of clinical and laboratory performance. Additionally, the system can generate reports required for external audits—such as RedLara's Annual Report—directly from the database, significantly reducing the administrative burden and ensuring accuracy.

### Step 7: Managing Customer Expectations

A well-implemented QMS aligns service delivery with patient needs and expectations.

**Figure 5:**
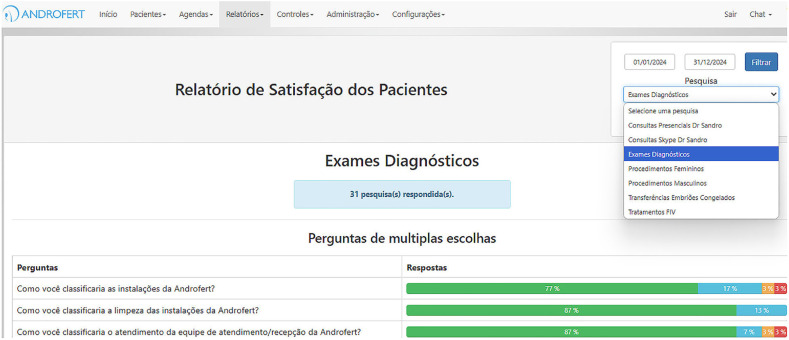
Illustrative Patient Satisfaction Survey (ClinisysIVF®).

- Patient Satisfaction Surveys: Regular surveys assist in identifying areas for improvement, ranging from clinical care to administrative efficiency (Figure-5).
- Employee Feedback and Engagement: Internal satisfaction surveys are conducted annually to evaluate staff morale and operational challenges. Insights from these surveys guide specific improvements in training, workflow efficiency, and team dynamics.

At ANDROFERT, patient satisfaction is continuously monitored as a key component of our Quality Management System using ClinisysIVF. The system automatically sends questionnaires via email to patients who have received care at our Fertility Center. Patients can log in to their personal ClinisysIVF portal to complete the surveys. Once submitted, responses are stored in the Cloud, and real-time reports are instantly generated for review by all relevant stakeholders. Customized reports can also be generated based on specific periods, procedures, or other applicable criteria.

## CASE STUDY: QMS IMPLEMENTATION AT ANDROFERT

The ANDROFERT clinic, a center of excellence in male reproductive health, began its QMS journey in 2006 with a homegrown ISO 9001-aligned model (33,39). Key milestones include (Figure-2):

### 1. Nonconformity and Corrective Action Registration

A core component of the QMS was establishing a system that enabled staff from any department to document nonconformities, even if they were not directly responsible for them. While training facilitated issue reporting, the biggest challenge was ensuring that corrective actions addressed root causes rather than just immediate problems. This analytical approach was essential for fostering a culture of continuous improvement.

### 2. Extensive Staff Training

A comprehensive training program was launched focusing on ethics, moral values, organizational principles, and teamwork. This initiative aimed to foster a sense of shared responsibility and collaboration among staff members, reinforcing the fundamental principles of quality management.

### 3. Team-Based Problem Solving

Nonconformities were assigned to departments instead of individuals to foster collaboration and eliminate a culture of blame. Teams were collectively responsible for addressing issues, strengthening professional relationships, and enhancing understanding of interdependence in delivering high-quality services. This approach required a significant cultural shift, encouraging staff to move beyond individual performance metrics and embrace a shared responsibility for outcomes.

### 4. Development of Standard Operating Procedures (SOPs)

Before implementing ISO 9001, SOPs primarily existed in laboratory operations. With the QMS rollout, SOPs expanded to encompass clinical and administrative activities, ensuring consistent procedures across all departments. This formalization improved staff training and boosted operational efficiency.

### 5. Quality Monitoring and Performance Metrics

A structured system for monitoring key performance indicators (KPIs) was implemented, and metrics such as fertilization rates and embryo development were tracked regularly. Routine meetings were held to analyze data, set improvement targets, and align performance with international benchmarks. This systematic review process ensured that quality enhancements were data-driven and sustained over time.

### 6. Transition to a Paper-Free Laboratory

Implementing an electronic information system (ClinisysIVF®, Clinisys, Brazil) allowed for real-time monitoring of quality metrics, decreasing manual workloads and enhancing accuracy. Each patient's data was incorporated into the system, enabling instant calculation of performance indicators and facilitating evidence-based decision-making.

Implementing the QMS was an evolving process requiring continuous adaptation and refinement. By incorporating ISO 9001 principles into daily operations, ANDROFERT successfully established a sustainable quality culture, strengthening its commitment to providing excellence in reproductive healthcare. The experience at ANDROFERT highlighted several critical lessons:

- Promoting a positive perspective on constructive criticism is crucial for ongoing improvement.
- Adopting a team-oriented approach to problem-solving promotes collaboration and accountability.
- Standardizing procedures across all operational areas ensures consistency and improves training effectiveness.
- Systematic quality monitoring supports data-driven decision-making and aligns clinical outcomes with global standards.

## BENEFITS OF A QMS IN ART CENTERS: ENHANCING MALE REPRODUCTIVE CARE

In vitro fertilization is an inherently complex process that involves professionals from multiple disciplines, including medicine, embryology, ultrasonography, nursing, and administration. Effective collaboration among these diverse specialists is essential to ensure safe, efficient, and integrated care. However, coordinating their interactions and maintaining consistency in service delivery present significant challenges for ART centers.

A well-implemented Quality Management System provides a robust structure to support excellence in all facets of ART. When adapted to incorporate male infertility services—diagnostics, microsurgical procedures, and specialized sperm handling—the QMS becomes a powerful tool to ensure safe, consistent, and evidence-based care.

The key benefits of a QMS tailored to male reproductive health are detailed in Table-2.

**Table 2:** Key Benefits of a Quality Management System Tailored to Male Reproductive Health

Index	Benefit Description
1	Improved diagnostic accuracy through standardized protocols in andrology, including hormonal assessment, genetic evaluation, and sperm DNA fragmentation testing.
2	Consistent surgical outcomes through microsurgical technique standardization (e.g., varicocele repair, micro-TESE), with postoperative audits to track complications and sperm retrieval success.
3	Optimized lab workflows for processing complex male samples, minimizing variability and maximizing fertilization potential.
4	Seamless interdisciplinary collaboration, integrating urologists, embryologists, reproductive endocrinologists, and andrologists through unified protocols and communication pathways.
5	Enhanced training and capacity building, especially relevant in academic partnerships like ours with UNICAMP (Department of Surgery, Division of Urology), where QMS-aligned supervision ensures safe and meaningful urology residents´ engagement.

QMS: quality management system; micro-TESE: microdissection testicular sperm extraction; UNICAMP: University of Campinas

## MEASURING QMS EFFECTIVENESS: A MALE-FOCUSED ART PERSPECTIVE

Effective quality management must be measurable. At our center, QMS performance is continuously evaluated through a combination of operational and clinical indicators, including those specific to male infertility services (Table-3).

**Table 3:** Measuring QMS Effectiveness in a Male-Focused ART Center.

Category	Description
Quality action logs	Tracking non-conformities, surgical complications, sperm retrieval rates, and protocol deviations.
Key performance indicators (KPIs)	Including embryologist- and surgeon-specific metrics adjusted by sperm origin (e.g., fertilization rates with testicular sperm).
Patient satisfaction data	Collected post-operatively and post-treatment, segmented by treatment pathway (e.g., micro-TESE + ICSI).
Internal benchmarking and external comparisons	Using data visualization tools (control charts, histograms, Pareto analyses) to identify trends and performance gaps.
Strategic management tools	SWOT analysis, balanced scorecards, and PDCA cycles support ongoing alignment with clinical goals and regulatory expectations.
Continuous improvement outcomes	Includes lower staff turnover and absenteeism, improved male patient trust and engagement, and higher procedural success rates.

SWOT – Strengths, Weaknesses, Opportunities, and Threats: A strategic planning tool used to identify internal and external factors that affect an organization's ability to achieve objectives. Strengths and Weaknesses are internal factors. Opportunities and Threats are external factors.PDCA – Plan-Do-Check-Act: A four-step iterative method for continuous process improvement. Plan: Identify a goal or process and plan changes. Do: Implement the planned change on a small scale. Check: Assess the results and determine effectiveness. Act: If successful, standardize the change; if not, refine and repeat.

Additionally, sustaining quality improvement requires ongoing evaluations, such as audits, self-assessments, management reviews, and structured discussions for improvement. Our experience shows that implementing a QMS enhances process control, reduces inefficiencies, and promotes a more organized and efficient work environment. This, in turn, leads to increased employee satisfaction, lower turnover, and minimal absenteeism—crucial factors for maintaining high standards in fertility care.

By systematically integrating QMS principles, ART Centers can achieve superior clinical outcomes, optimize patient experiences, and maintain high operational excellence.

## CONCLUSIONS

As ART becomes more complex and personalized, quality management systems are essential for delivering safe, effective, and patient-centered care. This is particularly true in the realm of male infertility, where success depends not only on technical expertise but also on multidisciplinary integration, individualized treatment, and a culture of continuous improvement. By implementing an ISO 9001-based QMS, our center has successfully embedded male reproductive health into every layer of care—from diagnostics and microsurgery to laboratory performance and patient support. Our partnership with the Division of Urology at UNICAMP has further expanded our capacity to train future specialists while upholding the highest quality standards. Ultimately, a well-structured QMS does more than streamline operations—it transforms an ART center into a cohesive, accountable, and excellence-driven institution. For programs dedicated to male reproductive health, this framework is not optional—it is essential.
